# Salivary oxidant/ antioxidant status and hematological parameters in patients with recurrent aphthous stomatitis

**Published:** 2016

**Authors:** Neda Babaee, Hamed Hosseinkazemi, Mahdi Pouramir, Oveis Khakbaz Baboli, Maede Salehi, Fatemeh Khadir, Ali Bijani, Mahsa Mehryari

**Affiliations:** 1 Department of Oral Medicine, Faculty of Dentistry, Babol University of Medical Sciences, Babol Iran.; 2 Cellular and Molecular Biology Research Center, Babol University of Medical Sciences, Babol, Iran.; 3 Department of oral and maxillofacial surgery, faculty of dentistry, Babol university of Medical Sciences, Babol Iran.; 4 Department of Oral Medicine, Faculty of Dentistry, Mazandaran University of Medical Sciences, Iran.; 5 Department of Biochemistry, Babol University of Medical Sciences, Babol, Iran.; 6 Social Determinant of Health Research Center, Babol University of Medical Sciences, Babol, Iran.; 7 Department of Oral Medicine, Faculty of Dentistry, Ardabil University of Medical Sciences, Ardabil, Iran.

**Keywords:** Recurrent aphthous stomatitis, Antioxidant, Saliva, Hematinic.

## Abstract

**Background:**

Recurrent aphthous stomatitis (RAS) is the most common inflammatory ulcerative condition of oral cavity. The aim of this study was to compare the levels of the salivary Malondialdehyde (MDA) and total antioxidant capacity (TAC) and blood parameter in RAS versus healthy controls.

**Methods:**

This case-control study consisted of 28 patients with RAS and 28 age and sex -matched control without RAS. Cell blood count was assessed by sysmex system, serum iron and total iron binding capacity was measured by standard laboratory kit and for ferritin ELISA kit was utilized. Salivary TAC and MDA level determined using FRAP and TBARS method respectively. Statistical analysis was performed using SPSS Version 21, chi-square test was used to compare proportions, and student’s t-test and Mann Whitney U-test were used for the comparison of quantitative variables

**Results:**

Salivary MDA level was significantly higher (p<0.001) and TAC level was significantly lower (p<0.042) in RAS as compared with the control group. Also, serum ferritin level was significantly higher in RAS patients (p<0.008).

**Conclusion:**

These findings indicate the alteration of oxidant/antioxidant status was observed in recurrent aphthous stomatitis, may be also associated with changing several hematinic parameters in this study. The finding maybe helpful to clarify the etiologies of RAS and possibely to improve the management or preventive options.

Recurrent aphthous stomatitis (RAS) is the most common inflammatory ulcerative condition of oral cavity, affecting up to 20% of population with three classic forms including minor, major and herpetiform. Despite numerous studies, the etiology of RAS has not been determined, but the abnormalities in several factors such as genetic, immunologic and hematological have been implicated. Other factors are local trauma, stress, hormonal alteration, nutrition, but at present, the most discussed topics of etiology and pathogenesis in RAS is oxidative stress (1-3). When the intracellular concentrations of reactive oxygen species (ROS) increase over the physiologic rates, oxidative stress may occur. It seems that the increase of oxidative stress may lead to imbalance of oxidant / antioxidant status which is crucial in inflammatory mechanism of RAS (1, 4, 5). Malondialdehyde (MDA) is the main product of lipid peroxidation and is found as an oxidative stress indicator. The human body has its own innate free radical scavengers, thus, it developed elaboration of antioxidant defense system that prevents or delays oxidative damage to target molecules. When it is insufficient, free radicals begin to damage the tissues and induce injuries to immune cells that finally increase the risk of infection and inflammation (5-8). 

Saliva as the first defense system of the body, has variant antioxidants. Each component of antioxidant system is effective for immune response, hence their activity all together coincide as a total antioxidant capacity (TAC) is more effective (9, 10). The prevalence of abnormal levels of hematological markers in RAS is 18-28%. Hematological parameters such as serum iron, total iron binding capacity (TIBC) and ferritin may have some contributions. However, variations in oxidant / antioxidant levels may have a etiologic and pathogenesis role through accelerating the formation of free radicals, so this may affect the balance of oxidant / antioxidant system (11-13). In accordance to limited studies about the evaluation of oxidant/ antioxidant system and hematological parameters in concurrent in RAS, the aim of this study was to evaluate both the salivary MDA and TAC and hematinic parameters in RAS patients. 

## Methods

All participants of this case-control study were recruited among patients presented to the Department of Oral Medicine Babol University of Medical Sciences, Babol, Iran. The protocol for this study was approved by the local research ethics committee. The objective of the study was explained to the 56 participants after giving their informed consent. There were 28 subjects in both case and control groups, age and sex-matched. All subjects were examined by an oral medicine expert and RAS was diagnosed clinically. The RAS group had oral ulcers while examining the history of RAS attack at least three times a year and all of them were minor aphthae. The control group was selected among patients without the presence or history of RAS. All of participants were not under a therapeutic regimen for the past 3 months and had not taken any drugs, smoked and consumed alcohol or any disease of the oral mucosa such as oral Lichen planus and periodontit and systemic disorders like behcet’s syndrome, crohn’s disease and etc (2, 3, 14).

**Saliva and blood samples: **The saliva and blood samples were obtained on the same day. Unstimulated saliva samples were collected between 9 to 11 by spitting method. The patients were asked not to eat, drink or brush 90 mins before the first sampling. The patients rinsed their mouths using distilled water. Then, they spitted into the plastic tubes one twice per min for 10 min. The samples were centrifuged at 2000g for 10 min at 4°C, the upper parts were drawn and stored in small aliquots at -20°C (1, 15). Fasting venous blood samples (5ml) were taken from each patient. The saliva samples were centrifuged at 3000g for 10 min at 4°C, and then analyzed for each parameter. Sysmex system was used to examine cell blood count and to assess serum iron and TIBC standard laboratory kit and for ferritin ELISA kit was used (8, 11).

**Measurement of MDA and TAC: **Salivary MDA was detected with thiobarbituric acid reactive substances (TBARS) method, reaction between MDA and TBA produced a pink pigment with a maximum absorption at 532 nm measured by spectrophotometry (16). Salivary TAC level was determined using a FRAP method (ferric reducing antioxidant power). The absorbance of the sample and standard solution in a wavelength of 593 nm were measured at 37°C. Iron (II) sulfate (125-250-500-1000µM) solutions were used as the standards. By comparing the absorbance of the sample and standards, concentration of antioxidants in the samples was obtained (8, 14).

**Statistical analysis: **Statistical analysis was performed using SPSS Version 21, chi-square test was used to compare proportions, and student’s t-test and Mann Whitney U-test were used for the comparison of quantitative variables 

## Results

**Fig 1:**
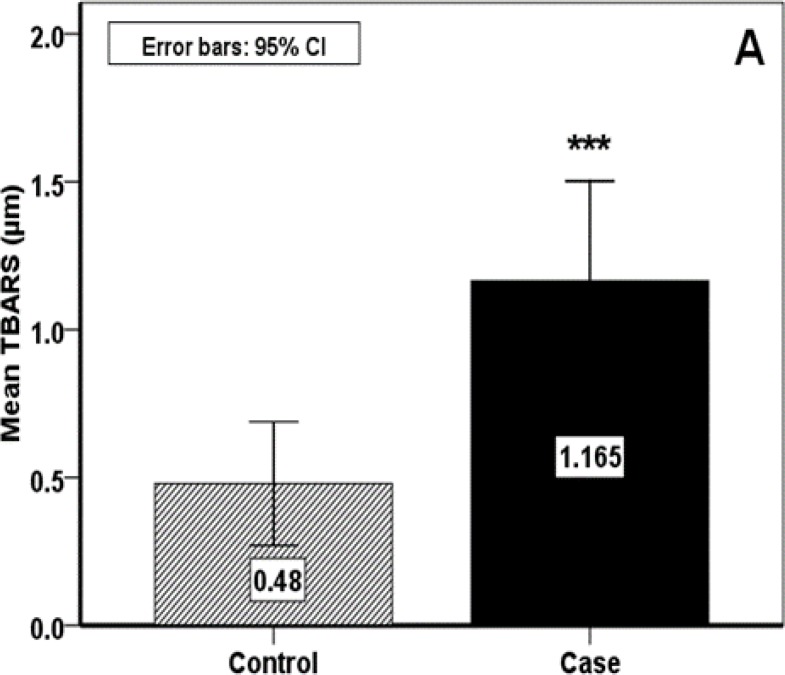
Salivary oxidant evaluation

**Fig 2:**
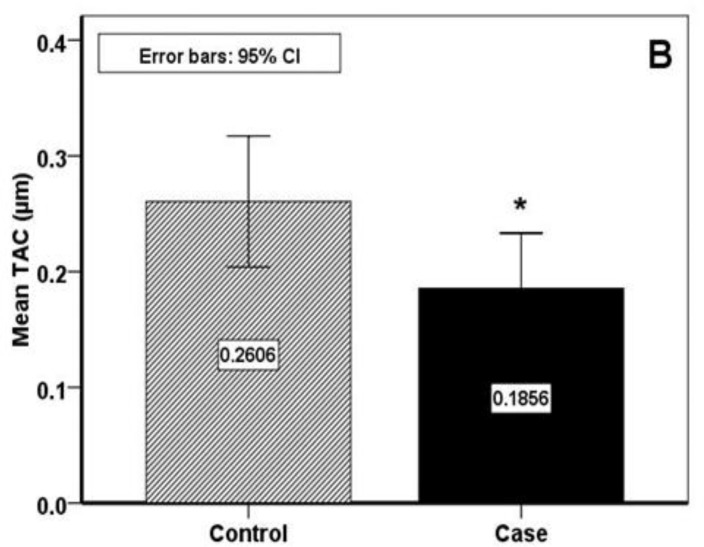
Salivary antioxidant evaluation

The RAS group included 13 (46.4%) females and 15 (53.6%) males with the mean age of 34.7±15.18 years. In the healthy group, there were 15 females and 13 males with the mean age of 32.03±11.30 years. The value of hematological parameters and salivary MDA and TAC are presented in table 1. With regard to oxidant / antioxidant status, salivary MDA level was significantly higher in RAS group (P<0.001) and TAC level was significantly lower (P<0.042) (fig 1, 2).

**Fig 3:**
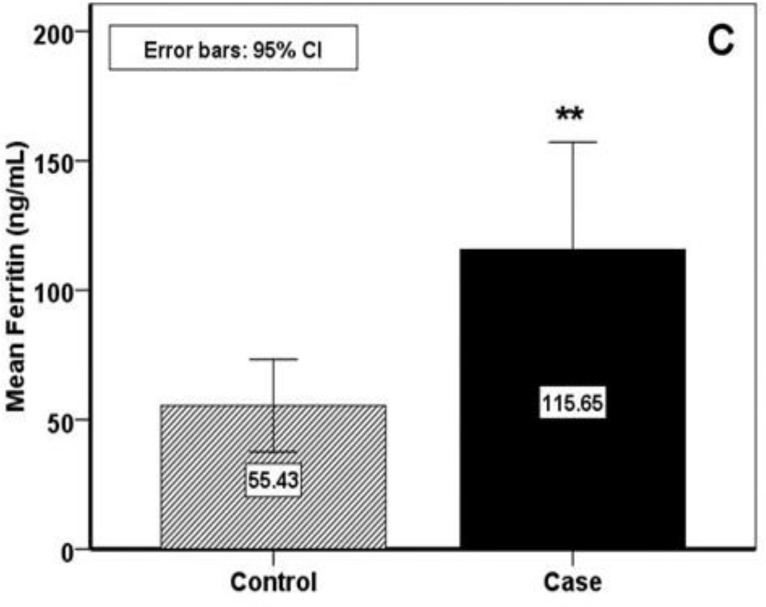
Serum ferritin evaluation

To compare the hematological parameters between 2 groups, the serum ferritin level was significantly higher in RAS patients (P<0.008) but SI and TIBC level had no significant difference in either group (fig 3). Variations in other blood parameters are shown in table 1. Red blood cell (RBC) and platelet level were significantly higher in RAS group (P<0.04 and P<0.000) while mean corpuscular value (MCV) and mean corpuscular (MCH) were significantly lower (P<0.002, P<0.033). Regarding the hemoglobin level, there was no significant difference between the control and the RAS group; however 9 (32.1%) patients in control group and 17 (60.7%) patients in RAS group had lower level of Hb than the normal range but there was no significant difference according to the gender. Also in RAS patients with lower level of Hb than normal range, ferritin was significantly higher (P<0.011) but in control group it was not observed (P<0.792).

**Table 1:** The value of salivary oxidant/antioxidant status and hematological parameters in control and case group of study

**Parameter**	**Control** **Mean±SD**	**Case** **Mean±SD**	**P-value**
WBC (cu.mm)	7917.85±11251.80	6071.42±1527.30	0.393
RBC (mil/cu.mm)	4.38±0.31	4.64±0.57	0.043
Hb (g/dl)	12.98±46.06	12.87±1.04	0.709
HCT (%)	39.24±2.37	38.50±3.29	0.340
MCV (fl)	88.77±6.71	82.58±7.21	0.002
MCH (pg)	29.37±2.37	28.02±2.23	0.033
MCHC (g/dl)	32.87±1.42	32.88±1.16	0.975
Platelete (1000/cumm)	207107.14±42080.46	276892.85±59541.66	0.000
SI (mg/dl)	75.00±33.63	75.25±28.17	0.976
TIBC (mg/dl)	334.60±27.86	330.92±36.46	0.673
Feritin (ng/ml)	55.42±46.06	115.64±107.00	0.008
MDA (µm)	0.47±0.53	1.16±0.86	0.001
TAC (µm)	0.26±0.14	0.18±0.12	0.042

WBC: White blood cell, RBC: Red blood cell, Hb: Hemoglobin, Hct: Hematocrit, MCV: Mean corpuscular volume, MCH: Mean corpuscular hemoglobin, MCHC: Mean corpuscular hemoglobin concentration

## Discussion

Recurrent apthous stomatitis (RAS) is the most common inflammatory ulcerative condition of oral cavity. Despite the broad research, the etiology of RAS remains obscure. Several factors seem to have a contribution in the etiology and pathogenesis of RAS. Some etiologic factors may have a direct or indirect effect on oxidant/antioxidant system. Also, the alteration of hematological parameters have been reported among the most significant factor for RAS (1, 2, 10, 17). 

In the present study, salivary MDA as an oxidant index was significantly higher in patients with RAS. Whereas, salivary TAC as an antioxidant marker showed significantly lower than the control. According to hematological parameters, the statistical analysis demonstrated significant difference in ferritin between case and control groups which was greater in RAS. 

Considerable activity of reactive oxygen radicals may lead to destructive cell functions and structural integrity. Oxidative stress in biological system may be induced by the overload of oxidant species or consumptions of antioxidant, have the antioxidant level has decreased. It is widely accepted that the imbalance between oxidant and antioxidant causes many inflammatory oral mucosa diseases from infections to immunologic diseases. Several studies have reported that the imbalance of oxidant/ antioxidant status is responsible for tissue damage in patients with RAS (4, 18, 19). MDA is one of the most frequently used indicators of lipid peroxidation, furthermore, the excess generation of MDA has the toxic effect that leads to changes of proteins, modifications of amino-acid chain, and lipid structure (20, 21). 

A decreased in antioxidant activity is one of the factors that play a role in the pathogenesis of RAS, measurement of TAC may reflect the antioxidant defense activity of the organisms accurately (19). Some researchers reported that no significant differences were found in salivary or serum TAC. They suggested that reactive oxygen species (ROS) may not play a role in the etiology of RAS. In addition, one interpretation can be that the oxidant / antioxidant system including both the variant enzymatic and non- enzymatic components, were not utilized equally in oxidative stress process. Hence, the total antioxidant capacity did not express noticeable declination in those studies (9, 14, 22).

Ferritin is one of the hematological parameters with various activities and is a main biomarker of iron stores that when reach to lower level than normal range indicates iron deficiency Reduced level of ferritin in association with low SI and high TIBC levels is presented as iron deficiency anemia. and higher than normal levels indicate high saturation of iron like hemochromatosis or iron overload. In addition, increased serum ferritin may also be a marker of chronic inflammation in many conditions like chronic hemodialysis. Elevated serum ferritin in RAS indicates as a marker of inflammatory process like erythrocyte sedimentation rate (ESR) or c-reactive protein (CRP) as seen in many chronic diseases, which is due to inflammatory cytokines (such as IL-1 and IFN-γ and hepcidin protein (23-26).

The results of the earlier studies regarding serum ferritin levels in RAS are conflicting. Since in several studies ferritin was investigated with iron deficiency, so it was shown low level in these patients or no significant differences (27-31). In recent study, there has not been significant differences of serum iron and TIBC between the two study groups indicating lack of association of ferritin that in dependently was higher in RAS. The important conclusion from the comparison of the previous and present study is that ferritin over-expression in RAS patients may explain the main role of its inflammatory mechanisms (21, 23, 25). Ferritin is a hematological parameter of iron stores of body that can accelerate oxidative damage. It has been detected that ferritin production can trigger peroxidation of some types of lipids. The generation of reactive oxygen species (ROS) is a steady - state cellular event in tissues that can induce a variety of pathophysiological conditions such as inflammation, immunological disorder, hypoxia and etc. (21, 24-26). ROS causes oxidative stress and often makes the damage of macromolecules (DNA, protein and lipid) potentially leading to cellular apoptosis. 

However, one of the most common etiologic factors of RAS is inflammatory and immunologic reactions include cytotoxicity T- lymphocytes to oral epithelium, antibody dependent to cell- mediated cytotoxicity and deficiency subgroups in lymphocyte (2-3). Another explanation of mechanism for increasing ferritin in RAS group may be due to a protective trait against oxidative stress by making chelation with free iron during increased oxidative stress. However, either inflammatory or protective mechanism of ferritin, the present study showed significantly higher level in RAS group (27). Further studies are needed to clarify the main reason of increasing level of ferritin. Several studies have suggested the importance of hemotological parameters in patients with RAS, whereas controversies exist (27-31). Our results showed that RBC and platelets had higher level in case group whereas MCV and MCH had lower level. Accordance to hemoglobin there was no significant difference between the case and control group, however in both groups Hb level was lower than the normal range. It is interpreted that excess free iron due to breakdown of Hb and unusual circulating of Hb in reticuloendothelial cells may lead to a positive feedback of ferritin synthesis. Also reduced Hb may predispose to additional gastrointestinal absorption of iron which is known as one of the major determinants of increasing ferritin level (21, 32-33). Consequently, the evaluation of hematinic parameters in RAS patients need to be studied extensively in many aspects and further studies. In conclusion, the alteration of oxidant/antioxidant status was observed in recurrent aphthous stomatitis, may be also associated with changing several hematinic parameters in this study. The finding maybe helpful to clarify the etiologies of RAS and possibely to improve the management or preventive options.

## Notes

**Funding** : This study was supported by a grant from the Vice-Chancellery of Research and Technology of Babol University of Medical Sciences (grant no. 9237522) and This study is a thesis project of Mahsa Mehryari (No: 80).
